# *It’s Not Just*: Evaluation of a Media Campaign to Motivate Action Around Targeting of Menthol Tobacco in Black Communities

**DOI:** 10.5888/pcd21.230237

**Published:** 2024-04-11

**Authors:** Matthew E. Eggers, James M. Nonnemaker, Lisa K. Kelly, Christina Ortega-Peluso, Elizabeth Anker, Jennifer Lee, OlaOluwa Fajobi, Nicole B. Swires

**Affiliations:** 1 RTI International, Research Triangle Park, North Carolina; 2 New York State Department of Health, Albany, New York

## Abstract

**Introduction:**

For more than 60 years, tobacco companies have aggressively marketed menthol tobacco products in Black communities. In 2021, New York State Department of Health–funded grantees launched a media campaign aimed toward civically engaged New York adults to educate and mobilize community action to prevent targeted marketing of menthol tobacco. This study examined audience reactions to the campaign and associations between campaign awareness and key outcomes.

**Methods:**

Following campaign implementation, we administered 2 online, cross-sectional surveys to 2,000 civically engaged New York adults to assess campaign awareness, audience reactions, and campaign-related attitudes and behaviors. We examined sociodemographic differences in audience reactions and assessed multivariate associations between campaign awareness and key outcomes.

**Results:**

Overall, 40% of respondents were aware of the campaign. Perceived advertisement (ad) effectiveness was higher among Black, Hispanic, and nonsmoking respondents and those aware of the campaign. Negative reactions to ads were higher at wave 1, among non-Hispanic White and male respondents, and among current smokers. Campaign awareness was positively associated with campaign-related beliefs. The association between campaign awareness and support for a menthol ban varied by survey wave and race, with positive associations at wave 2 and among non-Hispanic White respondents only. Among wave 2 respondents only, campaign awareness was positively associated with actions to reduce the targeting of menthol in Black communities.

**Conclusion:**

Media campaigns can play an important role in raising awareness of menthol tobacco product targeting in Black communities and building public support for local and statewide menthol restrictions that may be implemented before federal product standards are in place.

**Summary**

**What is already known on this topic?**

For more than 60 years, tobacco companies have aggressively marketed menthol tobacco products in Black communities.

**What is added by this report?**

A statewide media campaign to raise awareness of menthol tobacco targeting in Black communities resulted in moderate reach, with campaign messaging that was received favorably by priority audiences and with positive associations between campaign awareness and beliefs and behaviors the campaign sought to influence.

**What are the implications for public health practice?**

Media campaigns can play an important role in raising awareness of the impact of menthol tobacco product targeting in Black communities and building public support for local and statewide menthol restrictions that may be implemented before federal product standards are in place.

## Introduction

Tobacco use is the leading cause of preventable death and disease in the United States (1). Despite comparable rates of cigarette smoking prevalence between Black (14.4%) and White (13.3%) non-Hispanic adults (2), Black people disproportionately bear the burden of tobacco-related illness and death. Among all racial and ethnic groups in the US, Black people have the highest death rates for lung cancer, heart disease, hypertension, and stroke — all health conditions that have been linked to tobacco use (3).

Racial disparities in tobacco-related health outcomes may be driven in part by smoking menthol cigarettes. In the US and in New York City, 85% and 89% (respectively) of Black people who smoke cigarettes use menthol cigarettes (4,5). Menthol’s cooling properties can mask the harshness of cigarette smoke, which makes them easier to smoke and increases the likelihood of addiction (6,7). For more than 60 years, tobacco companies have aggressively marketed menthol tobacco products in Black communities (8). Neighborhoods with a higher proportion of Black residents have a higher number of tobacco retailers, more marketing of menthol tobacco products, and more tobacco marketing in general (9).

In 2022, the US Food and Drug Administration (FDA) proposed a rule prohibiting menthol as a characterizing flavor in cigarettes (10). In 2023, the New York State Executive Budget included legislation to end the sale of all flavored tobacco products, including menthol cigarettes (11). This provision was left out of the legislative budget and thus did not become New York law in 2023 (12); nevertheless, menthol restrictions continue to be a policy priority for the New York State Department of Health (NYSDOH). Nationally, approximately two-thirds (62.3%) of adults support policies to prohibit the sale of menthol cigarettes, and support is similar among non-Hispanic Black adults (61.5%) (13).

New York State’s proposed menthol legislation was preceded by sustained efforts from NYSDOH-funded grantees to advance tobacco-free norms in the state. As part of these efforts, in 2021 NYSDOH-funded Advancing Tobacco-Free Communities (ATFC) grantees worked collaboratively to develop the statewide *It’s Not Just* (INJ) media campaign to raise awareness of the impact of menthol tobacco use in Black communities and mobilize community action to prevent targeted marketing and sales of menthol tobacco. The campaign was aimed toward civically engaged New York adults and included digital video, print, and displays, digital radio, and social media spots; a statewide public relations campaign; and distribution of educational materials and talking points to support menthol ban advocacy efforts.

As menthol restrictions are being advanced by local communities, states, and the federal government, media campaigns like INJ can play an important role in building public support for such policies. The INJ campaign provides an opportunity to evaluate how media campaigns can influence beliefs and actions to counter tobacco marketing efforts. In this study, we conducted 2 surveys to assess audience reactions to and awareness of the INJ media campaign and examine associations between campaign awareness and key outcomes.

## Methods

### Campaign development and launch

The INJ campaign was developed collaboratively by ATFC grantees, NYSDOH, and Pinkney Hugo Group (PHG, the media vendor) in consultation with the Center for Black Health and Equity (CBHE). Before campaign launch, PHG conducted extensive pretesting of campaign materials via 2 separate surveys of the general adult population (N = 850) and Black adults (N = 811), balanced by region to ensure geographic representation across New York State. The pretesting surveys assessed receptivity, emotional reactions, and perceived likelihood of taking actions (eg, talking with family and friends, posting to social media, communicating with decision makers) in response to 2 advertisement (ad) concepts. Findings from the pretesting were used to select and refine the messaging concepts and final ad campaign materials. Measures used in the pretesting surveys (not described in detail in this article) were distinct from those used to evaluate the final executed media campaign messaging.

Campaign messaging featured Black people from communities targeted by the tobacco industry, along with voiceover and text describing the adverse impact of menthol tobacco in Black communities and links to educational and policy support resources. The priority audience for the media campaign was adult residents of New York State who were civically engaged, active participants of a community or church group, or educators or health care providers. These groups were identified as the priority campaign audience because they were groups hypothesized to be invested in their communities’ health and well-being, receptive to campaign messaging, and likely to take action in response to the campaign. Notably, the priority audience for campaign delivery (ie, civically engaged adults) and the audience featured in campaign messaging (ie, Black residents who have been targeted by the tobacco industry) are not mutually exclusive; the campaign sought to reach a broad audience of civically engaged adults — including but not limited to Black communities affected by tobacco industry targeting — who would likely have the ability to effect change around menthol tobacco policy.

The campaign launch on May 16, 2021, coincided with *No Menthol Sunday*, CBHE’s faith-based initiative, which provided a toolkit equipping participants with educational materials, strategies, and talking points to support policy action against menthol tobacco. The campaign aired statewide and included spots on iHeart radio (approximately 25% of ad budget), social media (ie, Facebook, Instagram, and Twitter: 19%), print (19%), digital television (14%), gas station televisions (11%), YouTube (6%), and digital display (6%), and it was also accompanied by a statewide ATFC public relations campaign assisted by PHG that included press releases and media pitches. The campaign has aired continuously since its launch and will run through June 2024. In tandem with the initial campaign iteration focused on menthol targeting in Black communities, the INJ campaign was also extended to reach other communities disproportionately affected by tobacco industry marketing, including youth and the LGBTQIA+ community.

### Study procedures

NYSDOH and RTI International, the organization conducting this research in partnership with NYSDOH, administered 2 online, cross-sectional surveys in June and July of 2021 (n = 1,000) and in August of 2022 (n = 1,000). The first survey wave was administered approximately 1 month after the media campaign launch, and the second survey wave was administered approximately 1 year later. (Due to evaluation resource limitations, a baseline [pre-exposure] survey was not feasible.) Participants from both survey waves were recruited from a non–probability-based web panel managed by Kantar (Bain Capital). The Kantar panel includes approximately 1.3 million consumers who are recruited on an ongoing basis via social media, online ads, and affiliate corporate networks.

Eligibility criteria for survey participation was aligned with the priority audience for the campaign. To be eligible, participants had to be an adult (aged 18 y or older) resident of New York State who met 1 or more of the following criteria: has contacted a public official or signed an online petition to express their opinion, attended a public meeting about community affairs, or worked with others to improve their community in the past year (adapted from Levine, 2012) (14); follows, engages with, or supports social cause accounts and campaigns on social media; is an active member of a civic organization (eg, YMCA), social justice movement, school parent–teacher association, environmental group, or religious organization; or is an educator in a K–12 school or is a health care provider. Little is known about the optimum exposure level and mix of channels or platforms to achieve detectable, population-level effects for largely digital media campaigns like INJ (15). Therefore, by aligning the survey recruitment with the priority population for the media campaign, we sought to ensure representation from groups prioritized in campaign delivery and maximize the potential to detect campaign effects with limited evaluation resources.

In addition to these eligibility criteria, we set quotas to ensure sufficient representation from key audience segments and facilitate cross-sociodemographic analyses. Specifically, we sought to maximize participation from people who identify as Black — communities of which are the subject of the campaign — and current smokers who would be most directly affected by actions to reduce the targeting of menthol tobacco products in Black communities. We also set quotas to achieve a balanced distribution across age groups (18–34 y, 35–54 y, and ≥55 y).

For each survey, panelists who had indicated in their panel profile that they met the age and geographic criteria were sent a study invite and directed to a brief screener survey to assess full study eligibility. After consenting to participate, eligible participants completed a 15-minute survey. Upon survey completion, participants received nonmonetary “points” that could be redeemed for online gift certificates, merchandise, or cash. The RTI institutional review board determined that this activity was conducted for evaluation purposes and thus did not meet the definition of research with human subjects.

### Measures

The surveys included the following key measures:

*Campaign awareness.* The media campaign included digital video, radio, and static image social media and banner ads. For each ad type, participants were shown or played an audio clip of the ad or a random selection of ads and asked if they had seen or heard the ad in the past 3 months. We created an indicator variable of campaign awareness that was coded 1 (“aware”) if any ads had been seen or heard and 0 (“not aware”) if no ads had been seen or heard.

*Perceived effectiveness *(PE). After viewing the digital video ad, participants were asked to indicate their agreement (1 being “strongly disagree” to 5 being “strongly agree”) with the following statements: “This ad is . . .” “worth remembering”; “grabbed my attention”; “is informative”; “is meaningful to me”; “is convincing”; or “is powerful” (16). We averaged scores from these 6 items to create a scaled PE measure with a range of 1 to 5 (mean [SD] = 3.79 [0.90]; α = 0.91).

*Negative reactions *(NR). For the digital video ad, we also asked participants to indicate their agreement (1 being “strongly disagree” to 5 being “strongly agree”) with the following items: “This ad is . . .” “phony”; “exaggerated”; “misleading”; or “deceptive” (17,18). We averaged scores from these 4 items to create a scaled measure of NR with a range of 1 to 5 (α = 0.90).

*Campaign-related beliefs.* We asked participants to indicate their agreement (1 being “strongly disagree” to 4 being “strongly agree”) with the following statements: “The tobacco industry heavily targets marketing of menthol-flavored tobacco products to African American/Black populations”; “There are more stores that sell tobacco in predominantly African American/Black neighborhoods compared to other neighborhoods”; “Most African American/Black smokers started by using menthol cigarettes”; “African American/Black communities have more advertising and cheaper prices for menthol cigarettes”; “The cooling flavor of menthol cigarettes makes them easier to get hooked on”; “Menthol cigarettes are harder to quit than nonmenthol cigarettes”; “Smoking-related illnesses are the number 1 cause of death for Black people.” We averaged scores from these 7 items to create a scaled measure of campaign-related belief endorsement with a range of 1 to 4 (α = 0.86).

*Support for a menthol cigarette ban.* We assessed support for a menthol cigarette ban with the following question: “What is your opinion about policies that ban the sale of menthol cigarettes? Are you . . . [1, “strongly against” to 5, “strongly in favor”].

*Actions to reduce tobacco targeting.* We asked participants whether they had taken any of the following actions in an attempt to reduce the targeting of tobacco products toward African American/Black communities in the past 3 months: “written to a local newspaper”; “signed a petition online”; “contacted an organization (such as New York Health Department, Tobacco Free New York State)”; “contacted an elected official”; “attended a meeting or joined an action group”; or “shared a petition on social media or by email.” We created an index representing the total number of actions taken, with a range of 0 to 6.

*Sociodemographic and geographic characteristics.* We also assessed race and ethnicity, age, sex, educational attainment, current use of menthol and nonmenthol cigarettes, and geographic region (New York City Designated Market Area [DMA] vs rest of state).

### Analysis

We calculated means, proportions, and frequencies for sociodemographic and geographic characteristics and campaign awareness, overall and by survey wave. To examine audience reactions to the media campaign, we first conducted 2 separate multivariable linear regressions of PE and NR each as dependent variables regressed on campaign awareness, survey wave, race and ethnicity, age, sex, educational attainment, smoking status, and geographic region. We then estimated model-predicted mean PE and NR scores, overall and across levels of each independent variable. To examine associations between campaign awareness and key campaign-related outcomes, we conducted 3 separate multivariable linear regressions of beliefs, policy support, and actions taken to reduce tobacco product targeting as dependent variables. Each model included campaign awareness, survey wave, race and ethnicity, and interactions of campaign awareness by survey wave and campaign awareness by race and ethnicity as primary independent variables. We included age, sex, educational attainment, current smoking status, and geographic region as control variables in each model. For models in which an interaction was significant, we estimated model-predicted mean dependent variable scores across each level of the interaction variable to aid in the interpretation of results. All analyses were conducted using Stata version 17.0 (StataCorp LLC).

## Results

Overall, 39.7% of respondents reported being aware of any campaign ad (35.6% at Wave 1 and 43.7% at Wave 2) (Table 1). Most respondents were non-Hispanic White (55.7%), female (65.3%), did not currently smoke cigarettes (72.4%), and resided in the New York City DMA (64.4%). The highest proportion of respondents were aged 55 years or older (32.9%) and had a bachelor’s degree or higher (44.9%).

**Table 1:** Campaign Awareness and Sociodemographic and Tobacco Use Characteristics of the Study Sample (N = 2,000), *It’s Not Just* Media Campaign, New York State, 2021–2022

Variable	Overall^a^	Wave 1	Wave 2
	No. (%)		
**Campaign awareness**			
Unaware of campaign ads	1,203 (60.3)	641 (64.4)	562 (56.3)
Aware of any campaign ads	791 (39.7)	355 (35.6)	436 (43.7)
**Race and ethnicity**			
Non-Hispanic White	1,110 (55.7)	590 (59.2)	520 (52.1)
Non-Hispanic Black	372 (18.7)	161 (16.2)	211 (21.1)
Other, non-Hispanic	128 (6.4)	73 (7.3)	55 (5.5)
Hispanic	384 (19.3)	172 (17.3)	212 (21.2)
**Age, y**			
18–24	384 (19.3)	219 (22.0)	165 (16.5)
25–34	306 (15.3)	138 (13.9)	168 (16.8)
35–44	367 (18.4)	127 (12.8)	240 (24.1)
45–54	281 (14.1)	139 (14.0)	142 (14.2)
≥55	656 (32.9)	373 (37.5)	283 (28.4)
**Sex**			
Female	1,295 (65.3)	631 (63.6)	664 (66.9)
Male	689 (34.7)	361 (36.4)	328 (33.1)
**Education**			
High school or less	456 (22.9)	234 (23.5)	222 (22.2)
Some college	642 (32.2)	319 (32.0)	323 (32.4)
Bachelor's degree or more	896 (44.9)	443 (44.5)	453 (45.4)
**Cigarette smoking**			
Does not currently smoke cigarettes	1,444 (72.4)	769 (77.2)	675 (67.6)
Currently smokes nonmenthol cigarettes only	165 (8.3)	83 (8.3)	82 (8.2)
Currently smokes menthol cigarettes	385 (19.3)	144 (14.5)	241 (24.2)
**Geographic region**			
Rest of state	710 (35.6)	356 (35.7)	354 (35.5)
New York City DMA	1,284 (64.4)	640 (64.3)	644 (64.5)

Abbreviation: DMA, designated market area.

a Numbers may not sum to the total overall (N = 2,000) or within-wave (n = 1,000 each) sample sizes due to missing responses for some variables.

Table 2 shows model-predicted mean PE and NR scores for the campaign video ad, overall and across select sample characteristics. Overall, mean PE was 3.79 and mean NR was 2.34. For context, mean scores can be compared with the response scales for the items that make up the PE and NR scales, with the mean PE score (score = 3.79) being close to “agree” and the mean NR score (score = 2.34) being between “neither agree nor disagree” and “disagree.” Mean PE was higher among respondents previously aware of the campaign (score = 3.99) compared with those who were not (score = 3.66), among non-Hispanic Black (score = 3.97) and Hispanic (score = 3.87) respondents compared with non-Hispanic White respondents (score = 3.72), and among respondents aged 35 years or older (score = 3.82–3.91) compared with respondents aged 18 to 24 (score = 3.59). Mean PE was lower among respondents who currently smoked nonmenthol cigarettes only (score = 3.46) compared with those who did not currently smoke cigarettes (score = 3.84). Mean NR was lower among wave 2 respondents (score = 2.26) than wave 1 respondents (score = 2.43) and among non-Hispanic Black (score = 2.12) versus non-Hispanic White (score = 2.42) respondents. Mean NR was higher among male (score = 2.45) versus female (score = 2.29) respondents and among those who currently smoked nonmenthol (score = 2.72) and menthol (score = 2.57) cigarettes versus nonsmokers (score = 2.24).

**Table 2:** Model-Predicted Mean Perceived Effectiveness and Negative Reactions to Video Ad, Overall and by Select Sample Characteristics (N = 1,984)^a^, *It’s Not Just* Media Campaign, New York State, 2021–2022

Variable	Perceived effectiveness (range, 1–5)		Negative reactions (range, 1–5)	
	Mean (95% CI)	*P* value	Mean (95% CI)	*P* value
Overall	3.79 (3.75–3.83)	—	2.34 (2.30–2.39)	—
**Campaign awareness**				
Unaware of campaign ads (reference)	3.66 (3.61–3.72)	—	2.32 (2.26–2.39)	—
Aware of any campaign ads	3.99 (3.92–4.05)	<.001	2.38 (2.30–2.45)	.34
**Survey wave**				
Wave 1 (reference)	3.77 (3.71–3.82)	—	2.43 (2.37–2.49)	—
Wave 2	3.82 (3.76–3.87)	.21	2.26 (2.20–2.32)	<.001
**Race and ethnicity**				
Non-Hispanic White (reference)	3.72 (3.67–3.78)	—	2.42 (2.36–2.48)	—
Non-Hispanic Black	3.97 (3.88–4.07)	<.001	2.12 (2.01–2.22)	<.001
Other or multi-race, non-Hispanic	3.64 (3.48–3.79)	.31	2.35 (2.17–2.53)	.49
Hispanic	3.87 (3.77–3.96)	.01	2.35 (2.24–2.46)	.32
**Age, y**				
18–24 (reference)	3.59 (3.49–3.69)	—	2.47 (2.36–2.59)	—
25–34	3.66 (3.56–3.76)	.30	2.48 (2.37–2.60)	.93
35–44	3.88 (3.79–3.98)	<.001	2.47 (2.36–2.58)	.95
45–54	3.82 (3.72–3.92)	.002	2.37 (2.25–2.49)	.24
≥55	3.91 (3.83–3.98)	<.001	2.12 (2.04–2.21)	<.001
**Sex**				
Female (reference)	3.82 (3.78–3.87)	—	2.29 (2.23–2.35)	—
Male	3.73 (3.67–3.80)	.03	2.45 (2.37–2.52)	.002
**Educational attainment**				
High school or less (reference)	3.79 (3.71–3.87)	—	2.36 (2.27–2.46)	—
Some college	3.73 (3.66–3.80)	.30	2.34 (2.26–2.42)	.73
Bachelor's degree or more	3.84 (3.78–3.90)	.32	2.34 (2.27–2.41)	.70
**Cigarette smoking**				
Does not currently smoke cigarettes (reference)	3.84 (3.80–3.89)	—	2.24 (2.19–2.30)	—
Currently smokes non-menthol cigarettes only	3.46 (3.32–3.59)	<.001	2.72 (2.56–2.87)	<.001
Currently smokes menthol cigarettes	3.75 (3.66–3.85)	.11	2.57 (2.46–2.68)	<.001
**Geographic region**				
New York City DMA (reference)	3.82 (3.77–3.87)	—	2.34 (2.28–2.40)	—
Rest of state	3.74 (3.67–3.80)	.05	2.35 (2.28–2.43)	.76

Abbreviations: —, not applicable; DMA, designated market area; NR, negative reaction; PE, perceived effectiveness.

a Mean PE and NR scores were predicted from separate multivariable linear regressions of PE and NR each as dependent variables regressed on campaign awareness, survey wave, race and ethnicity, age, sex, educational attainment, smoking status, and geographic region. *P* values are based on *t* tests from these models.

Campaign-related beliefs were higher among respondents who were aware of campaign ads compared with those who were not aware (ß = 0.26, *P* < .001) and among those who were non-Hispanic Black (ß = 0.16, *P* = .002), non-Hispanic other or multi-race (ß = 0.18, *P* = .02), and Hispanic (ß = 0.17, *P* = .006) compared with non-Hispanic White respondents (Table 3).

**Table 3:** Linear Regressions of Campaign-Related Beliefs, Policy Support, and Action Index (N =1,984)^a^, *It’s Not Just* Media Campaign, New York State, 2021–2022

Independent variable	Belief scale (range, 1–4)		Policy support (range, 1–5)		Action index (range, 0–6)	
	ß (95% CI)	*P* value	ß (95% CI)	*P* value	ß (95% CI)	*P* value
**Campaign awareness**						
Not aware of any campaign ads	1 [Reference]					
Aware of any campaign ads	0.26 (0.15 to 0.37)	<.001	0.10 (−0.12 to 0.32)	.36	−0.02 (−0.23 to 0.18)	.84
**Survey wave**						
2021	1 [Reference]					
2022	−0.01 (−0.08 to 0.06)	.71	−0.05 (−0.20 to 0.09)	.47	−0.86 (−0.93 to −0.78)	<.001
**Race and ethnicity**						
Non-Hispanic White	1 [Reference]					
Non-Hispanic Black	0.16 (0.06 to 0.27)	.002	0.05 (−0.16 to 0.26)	.65	0.03 (−0.11 to 0.17)	.66
Other or multi-race, non-Hispanic	0.18 (0.03 to 0.33)	.02	0.04 (−0.27 to 0.35)	.79	0.09 (−0.10 to 0.29)	.34
Hispanic	0.17 (0.05 to 0.28)	.006	−0.04 (−0.28 to 0.20)	.75	0.37 (0.15 to 0.59)	.001
Campaign awareness*survey wave	0.08 (−0.03 to 0.19)	.17	0.49 (0.26 to 0.72)	<.001	1.59 (1.34 to 1.83)	<.001
Campaign awareness*race and ethnicity						
Campaign awareness*Black, non-Hispanic	−0.03 (−0.18 to 0.12)	.72	−0.36 (−0.67 to −0.05)	.02	0.15 (−0.18 to 0.49)	.37
Campaign awareness*other or multi-race, non-Hispanic	−0.07 (−0.30 to 0.17)	.57	−0.23 (−0.71 to 0.25)	.34	0.1 (−0.46 to 0.66)	.73
Campaign awareness*Hispanic	−0.11 (−0.27 to 0.04)	.16	−0.1 (−0.42 to 0.22)	.53	0.27 (−0.09 to 0.63)	.14

a Results presented are from separate multivariable linear regressions of scaled campaign-related beliefs, policy support, and an index of actions taken to reduce menthol targeting in Black communities, each as dependent variables regressed on the independent variables listed. *P*-values are based on *t* tests from these models.

**Figure 1:**
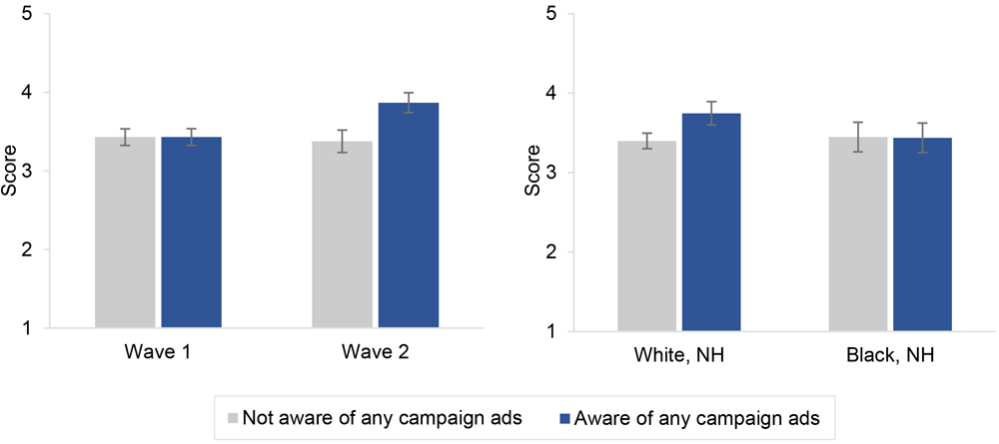
Model-predicted mean support for a menthol ban (score range, 1 = strongly against to 5 = strongly in favor), by campaign awareness and survey wave and race and ethnicity (N = 1,984), *It’s Not Just* media campaign, New York State, 2021. Campaign awareness was compared between respondents from waves 1 and 2 (panel A) and between non-Hispanic White and non-Hispanic Black respondents (panel B). Mean policy support scores were predicted from a multivariable linear regression with policy support as the dependent variable and campaign awareness, survey wave, race and ethnicity, and interactions of campaign awareness by survey wave and campaign awareness by race and ethnicity as primary independent variables (the model also included age, sex, educational attainment, current smoking status, and geographic region as control variables). Abbreviation: NH, non-Hispanic.

The association between campaign awareness and policy support varied by survey wave (ß = 0.49, *P* <.001) and non-Hispanic Black race (ß = −0.36, *P* = .02). To aid in the interpretation of these interactions, Figure 1 shows model-predicted mean policy support, by campaign awareness, survey wave, and race and ethnicity. Among wave 1 respondents, policy support was identical between respondents who were aware of and not aware of the campaign (mean = 3.43); among wave 2 respondents, policy support was higher among those aware of the campaign (mean = 3.87) compared with those not aware of the campaign (mean = 3.38) (Panel A). Among non-Hispanic White respondents, policy support was higher among those aware of the campaign (mean = 3.75) compared with those not aware of the campaign (mean = 3.40); among non-Hispanic Black respondents, policy support was similar between those aware of (mean = 3.44) and not aware of the campaign (mean = 3.45) (Panel B).

**Table:** 

Variable	Not aware of any campaign ads	Aware of any campaign ads
	Mean score (range, 1–5)	
**Wave**		
1	3.43	3.43
2	3.38	3.87
**Race and ethnicity**		
NH White	3.40	3.75
NH Black	3.45	3.44

**Figure 2:**
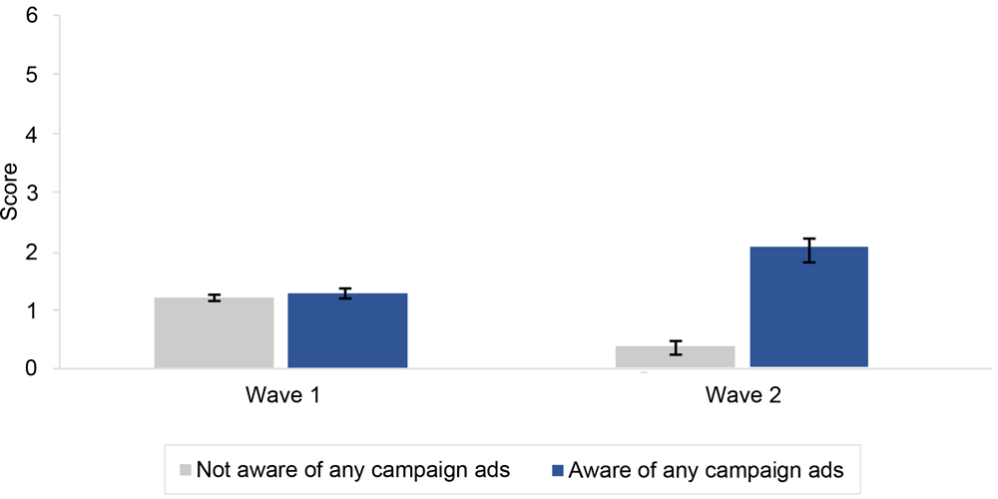
Model-predicted mean number of actions taken to reduce tobacco targeting in Black communities (range, 0–6), by campaign awareness and survey wave (N = 1,984), *It’s Not Just* media campaign, New York State, 2021. Mean action index scores were predicted from a multivariable linear regression with number of actions taken as the dependent variable and campaign awareness, survey wave, race and ethnicity, and interactions of campaign awareness by survey wave and campaign awareness by race and ethnicity as primary independent variables (the model also included age, sex, educational attainment, current smoking status, and geographic region as control variables).

Compared with non-Hispanic White respondents, Hispanic respondents had higher scores on the action index (ß = 0.37, *P* = .001). The association between campaign awareness and action index scores varied by survey wave (ß = 1.59, *P* <.001). Action index scores were similar between those aware (mean = 1.30) and not aware of the campaign (mean = 1.23) in wave 1 and were higher among those aware of the campaign (mean = 2.03) versus not aware in wave 2 (mean = 0.38) (Figure 2).

**Table:** 

Variable	Not aware of any campaign ads	Aware of any campaign ads
	Mean number of actions taken (range, 1–6)	
**Wave**		
1	1.23	1.30
2	0.38	2.03

## Discussion

We assessed awareness of and reactions to a media campaign to educate and motivate action around the targeting of menthol tobacco in Black communities and examined associations between campaign awareness and key outcomes the campaign sought to influence. Study findings demonstrated that the campaign resulted in moderate reach that increased over time, with nearly half of respondents reporting having seen any of the ads after approximately 1 year on air. Campaign awareness was below Centers for Disease Control and Prevention recommendations for mass-reach media campaigns to reach 75% of the priority audience (19), although no comparable benchmarks exist for primarily digital campaigns like INJ (15). We found that PE was higher among those who reported previous campaign exposure and that NR decreased between survey waves, suggesting that the campaign is being received more favorably as its reach and duration are extended. As the INJ media campaign continues amid a shifting menthol tobacco policy landscape, ongoing monitoring of awareness of and reactions to campaign messaging may be warranted.

A notable feature of the INJ campaign is that the priority audience for campaign delivery (ie, general population, civically engaged residents) is not necessarily the same as the audience depicted in the campaign (ie, Black communities); this incongruity could result in unintended consequences if the campaign is not well-received among the communities it is attempting to help. However, results from this study demonstrate that, across racial and ethnic groups, Black respondents perceived the campaign messaging to be most effective and had the lowest NR to messaging. This promising finding speaks to the robust community engagement underlying the campaign’s development and implementation, including consultation with CBHE, extensive pretesting with diverse groups, and accompanying public relations outreach to complement and reinforce the campaign’s messaging.

In examining differences in audience reactions by sociodemographic characteristics and tobacco use behaviors, a few additional patterns emerged. Our findings were consistent with previous research that demonstrates stronger self-reported negativity and defensive processing toward anti-tobacco messaging among smokers than nonsmokers (20). NR to campaign messaging were stronger among current menthol and nonmenthol smokers compared with nonsmokers. In contrast, menthol smokers perceived the messaging’s effectiveness at a level similar to nonsmokers. We also found that favorable reactions were generally positively correlated with age and that female participants had fewer NR than male participants, suggesting room for improvement in messaging to younger and male audiences who viewed the campaign less favorably than their counterparts.

Results from analyses of the association between campaign awareness and outcomes varied. After controlling for sociodemographic and geographic characteristics and tobacco use behaviors, we found that campaign awareness was associated with stronger endorsement of campaign-related beliefs with main effects that were robust across race and ethnicity and survey wave. Partially contrasting this result, the number of actions taken to reduce tobacco targeting in Black communities was also greater among those aware of the campaign, although this effect was only observed in the second survey wave. This pattern is consistent with theories of behavioral prediction (eg, theory of planned behavior) that posit that beliefs precede intentions and behavior (21). Our findings suggest that the campaign may have had a more immediate influence on beliefs, with downstream effects on behavioral actions commensurate with increased campaign duration and reach.

In contrast with the patterns above, we found that the association between campaign awareness and support for a menthol cigarette ban increased over time and varied by race and ethnicity, with support being higher among non-Hispanic White respondents who were aware of the campaign compared with those who were not; we found no difference in support between non-Hispanic Black respondents by campaign awareness. One possible explanation for the lack of difference in policy support by campaign awareness among Black respondents is that the issues depicted in the campaign ads may be less novel to Black communities, who have been centered in public discourse around a menthol ban. Black individuals who have been disproportionately burdened by tobacco industry marketing may have already solidified their opinions about a menthol ban, with little room to move resulting from campaign exposure. Previous public opinion research among a nationally representative panel of adults has shown majority support for a menthol ban across racial groups (13), although less is known about the extent to which public opinion has shifted since FDA’s proposed rulemaking in April 2022.

Another potential contributing factor to the racial and ethnic differences in association between campaign awareness and support for a menthol ban is the controversial nature of the topic. The potential public health benefits of a ban on menthol cigarettes are well established (22,23), and support for the federal ban is shared widely across national social justice and advocacy organizations, including the National Association for the Advancement of Colored People, the CBHE, and most of the Congressional Black Caucus (24). Nevertheless, FDA’s proposed ban on menthol cigarettes has been criticized as inherently paternalistic (25), while the American Civil Liberties Union has raised concerns that a menthol ban may lead to an illicit market of menthol cigarette sales that could exacerbate racial disparities in law enforcement (26). Results from this study suggest that although the influence of the INJ media campaign on related beliefs and actions is robust across racial and ethnic groups, the potential effect of the campaign on menthol policy support is more nuanced and perhaps reflects the polarization among Black communities around the topic of a potential menthol ban.

This study is subject to several limitations. First, data were collected using a convenience panel of adult New York State residents with a recruitment focused on civically engaged adults who were the priority audience for the campaign; as such, results may not be representative of adults in New York State in general, and the campaign may have been received differently among those not in the priority audience. Second, campaign awareness was assessed via self-report using aided recall methods, which may be subject to recall or social desirability bias that could lead to artificially inflated campaign awareness relative to estimates using unaided recall methods. Finally, the surveys were cross-sectional, and both waves were conducted following campaign implementation, which precludes an assessment of the temporality of campaign exposure and campaign-related beliefs and actions. Because a baseline or pre-exposure survey was not feasible (due to resource limitations), we cannot determine whether campaign-related outcomes were caused by campaign exposure or if those with favorable outcomes were more likely to recall the campaign due to preexisting beliefs and attitudes that aligned with campaign messaging.

Anti-tobacco media campaigns have been shown to increase cigarette smoking cessation attempts, reduce youth smoking initiation, and reduce smoking prevalence (27–29), but less is known about the effect of media campaigns aimed at increasing public understanding and support for policy changes (15). To our knowledge, our study is the first to evaluate a media campaign to educate and motivate action around the targeting of menthol tobacco in Black communities. It demonstrates that the INJ campaign resulted in moderate reach, with campaign messaging that was received favorably by priority audiences and with positive associations between campaign awareness and key campaign-related beliefs and behaviors.

The INJ campaign coincides with FDA’s recent announcement that it intends to advance product standards to ban menthol cigarettes. Results from a recent simulation study estimate that such a ban would result in a 15% reduction in cigarette smoking, reducing cumulative smoking- and vaping-attributable deaths by 650,000 over a 40-year period (30). Despite these anticipated public health benefits, the timeline for implementation of federal product standards is unknown and will likely be impacted by tobacco industry litigation. Our findings suggest that community education campaigns can play an important role in raising awareness of the impact of menthol tobacco product targeting in Black communities and building public support for local menthol restrictions that may be implemented before federal product standards are in place. Future research could evaluate additional INJ campaign iterations focusing on other communities disproportionately affected by tobacco industry marketing including youth and the LGBTQIA+ community.
